# Linguistic Markers of the Emotion Elaboration Surrounding the Confinement Period in the Italian Epicenter of COVID-19 Outbreak

**DOI:** 10.3389/fpsyg.2020.568281

**Published:** 2020-09-18

**Authors:** Attà Negri, Giovanbattista Andreoli, Arianna Barazzetti, Claudia Zamin, Christopher Christian

**Affiliations:** ^1^Department of Human and Social Sciences, University of Bergamo, Bergamo, Italy; ^2^Italian Society of Relationship Psychoanalysis, Milan, Italy; ^3^City College, City University of New York, New York, NY, United States

**Keywords:** expressive writing, emotion elaboration, writing and health, referential activity, linguistic measures, COVID-19

## Abstract

The expressive writing method has rarely been proposed in contexts of large-scale upheavals that affect large populations. In this study this method was applied as an intervention and tool of investigation during the confinement period in the Lombardy region, the Italian Epicenter of COVID-19 outbreak. Sixty-four participants took part in an online expressive writing project, and a total of 167 writings were collected together with some self-report evaluations on emotions and physical sensations. A linguistic analysis through two different sets of computerized linguistic measures was conducted on the collected writings in order to study the linguistic markers of emotion regulation and elaboration. Results indicated that online expressive writing has helped respondents to get more in touch with the intense emotions that were experienced following the upheavals they witnessed. Writing even only once or twice helped, particularly those respondents who had at least one COVID-19 patient among close friends or relatives. Their writings showed an intense emotional involvement together with the ability to reflect and reorganize the personal meaning of the events and emotions experienced. This study shows that expressive writing can be used in the context of a psychological emergency, both as a powerful instrument to investigate and detect the complex psychodynamic processes underpinning the distress, and as a useful intervention to reduce the negative impact of traumatic events.

## Introduction

In late February 2020, Italy suddenly discovered itself overrun by the Coronavirus Disease 2019 (COVID-19) outbreak. It was the first country in Europe to witness the rapid spread of the virus. The population was hit by feelings of great uncertainty and fear, compounded by the lack of knowledge about the virus’ lethality and confusion and about the effective measures to be taken to counter it. This happened in a particularly marked way in the city and surrounding areas of Bergamo and across the Lombardy region, which became the Italian epicenter of the epidemic, with about 40% of all confirmed cases and about 50% of all deaths in Italy. Within 2 weeks from the discovery of the first confirmed COVID-19 case, the Italian Government decided to impose a mandatory confinement at home for the entire Italian population as a preventive measure, a legal disposition never taken previously in Italian history. The strict confinement began on March 9, 2020 and gradually was decreased beginning May 4, 2020. Subsequently, most European countries adopted similar restrictions within a few weeks following the discovery of their first confirmed cases.

We can say thus that the COVID-19 pandemic and the consequent adopted confinement measures marked the greatest worldwide health, psychological, and economic crisis since the second World War. Millions of people were quarantined, hospitals were overwhelmed, experts warned of healthcare collapse, riots and protests broke out in various places, including prisons; countless people lost their jobs, and there were growing concerns of food shortages. Gloves and surgical masks, used as a barrier to viral transmission, were selling out (Rubin and Wessely, 2020). As we write this, the dire situation is still far from a complete resolution. It is thus evident that the current situation may be experienced as threatening not only from a physical health point of view, but also from a psychological one.

The situation generated by the COVID-19 outbreak can in all respects be considered as a psychological emergency for people. A psychological emergency can follow, not only a single traumatic event but can also be associated with a much more complex context, such as the one created by a pandemic (Goldmann and Galea, 2014). People in fact (a) experienced a real or perceived threat, (b) felt a strong pressure to act and make quick decisions, (c) sensed not having enough resources to deal with the threat, and (d) experienced a set of negative and intense emotions. A growing number of empirical studies is investigating this psychological impact. Personnel of COVID-19-dedicated healthcare settings, hospitalized or quarantined infected patients, relatives unable to visit in hospital or accompany to death their loved ones were the most exposed to the risk of this traumatic impact (Folkman and Greer, 2000). However, the general population also experienced a psychological emergency due to the non-stop stream of frightening information carried by news outlets, and in response the government-enforced measures of confinement. Most of the studies about the epidemics’ psychological effects document a series of negative symptoms including distress (Al-Rabiaah et al., 2020), post-traumatic stress disorder (Hawryluck et al., 2004), anxiety (McAlonan et al., 2007), depressive syndromes and anger (Dell’Osso et al., 2011), worries about the effects of quarantine and contagion on relatives, colleagues and friends (Maunder et al., 2003, 2006), abuse of alcohol and tobacco (Morganstein et al., 2017), shame and guilt (Van Bortel et al., 2016). Negative emotions are experienced by individuals especially during the closure of schools and businesses (Hall et al., 2008) and can have long-term psychological implications (Lee et al., 2007). Wang et al. (2020) highlighted that during the initial phase of COVID-19 outbreak in China, more than half of the respondents in a research study, rated the psychological impact as moderate-to-severe, and about one-third reported moderate-to-severe anxiety. Also dysfunctional behavioral reactions to the perceived threat have been registered, ranging from denial of fear to real panic attacks (Xiang et al., 2020). Moreover, the physical distancing required by confinement measures increased feelings of loneliness and exacerbated the eventual relational and mental health problems that may have been preexisting conditions prior to the pandemic.

Recent research has aimed to investigate and describe in depth the processes underpinning psychological symptoms and generic psychological distress registered during quarantine or the period marked by the outbreak. In particular, this research has focused on the intense emotional arousal and the associated defensive or dissociative processes surrounding the COVID-19 outbreak. Porcelli (2020), for example, has speculated that anxiety and fear are adaptive emotions that can protect the integrity and unity of the self. Scalabrini et al. (2020) argued that the fear of being infected or infecting others undermines the implicit and automatic process of anchoring the self to the world, leading to a collapse of an intersubjective connection and thus to existential anxiety and anguish. Schimmenti et al. (2020) analyzed the bodily, interpersonal, cognitive and behavioral manifestations of fear triggered by the COVID-19 outbreak. During a pandemic people typically become hypervigilant about any bodily change that might suggest an infection (fear of the body), try to protect the body as a treasure that may be lost (fear for the body), experience themselves as being potentially dangerous to their loved ones (fear for significant others), and experience significant others as potential threats (fear of significant others). Venuleo et al. (2020) described how the heightening uncertainty caused by the COVID-19 pandemic leads to immediate, black-and-white thinking and generalized interpretations of reality, at the cost of more analytical thought.

It is evident that empirically studying these complex psychodynamic processes underlying the experience associated with the COVID-19 pandemic is not a simple endeavor. They need to take into account conscious and unconscious reactions of individuals; familial and interpersonal transactions; and the social and communication processes, which are particularly powerful in the context of the profound uncertainty generated by a pandemic. One way to investigate these psychodynamic emotional processes is to study the language used by people in describing their experience. Language can be considered as a catalyzer of the emotional processing during the pandemic period performed by the body-mind-environment system. An example of the centrality of language in the construction, amplification, magnification, modulation and, hopefully the elaboration of emotions can be found in the ways in which social and mass media were used by people, journalists, politicians, scientists and governments during the outbreak. The type, quantity, credibility, context and form in which information was given heavily influenced the emotional experience of millions of people. The increase in the amount of information and misinformation broadcasted, which normally characterizes the initial phase of a pandemic, can produce an inevitable emotional over-reaction if it is not adequately managed (Loveday, 2020). It has been shown (Strong, 1990) that the language and the images used to describe the phenomenon of infection have an effect on people’s psychological reactions. For example, words like “war,” “killer,” “battle,” “invisible threat” contribute in fueling the negative feeling people can have in defining the danger (Wald, 2008).

Studying language also has the advantage of making possible the development of measures that tap the elaborative or dissociative processes used by people with respect to the emotions they are experiencing. This study takes as a point of reference the theories and research of Bucci (1997, 2013) and Pennebaker (1982, 2011), which connect the linguistic qualities of speech to specific psychological and interpersonal processes.

Bucci (1997, 2013) (Maskit and Murphy, 2011; Maskit, 2012; Mariani et al., 2013, 2020; Negri et al., 2019) have developed computerized linguistic measures of what they call the “referential process,” which is based on Multiple Code Theory (MCT). The referential process is the ability of human beings to translate the continuous and indistinct flow of emotional and sensorial experience into images and words, thus making it mentally manageable and communicable to others. It is not possible to completely translate all emotional and sensory experience into words. There is, therefore, always a certain amount of disconnection between what a person feels and what s/he can think and communicate of this experience. Such a disconnection could increase when the person experiences painful events and emotions, since some distancing process of painful sensations are activated. The greater the disconnection, the more the person feels emotional arousal without being able to mentally manipulate and communicate the experience being aroused. Interpersonal communication can reactivate the referential process. Normally the interpersonal communication that is able to reactivate the referential process proceeds from an emotion arousal phase to a symbolization one in which the emotional experience is put into images and words, and finally passes to a phase of reorganization and reflection that expands the meanings attributed to the experience. The computerized linguistic measures developed by Bucci and colleagues are able to detect all three phases described (emotional arousal, symbolization, and reorganization) and therefore to measure the emotional elaboration process put in place by a speaker or a writer.

Pennebaker (1982, 2011), Pennebaker and Smyth (1997), and Pennebaker and Evans (2014) developed a well-known research paradigm based mainly on the study of language contents and styles. Expressive writing is the principal method used by Pennebaker; it consists of asking people to write about their emotions and thoughts in at least three consecutive sessions. Several studies (Pennebaker and Beall, 1986; Booth et al., 1997; Smyth et al., 1999; Rosenberg et al., 2002; Stanton and Danoff-Burg, 2002; Gallant and Lafreniere, 2003; Frisina et al., 2004; Norman et al., 2004; Baikie et al., 2012; Doherty and Wenderoth, 2017) demonstrated that expressive writing can bring many important benefits, such as a reduction in medical visits, an increase of immune defenses, a reduction in drug use, a reduction of pain intensity level, better school and academic grades, general sensation of better well-being, a reduction in feelings of depression and anger, a decrease in time to find a new job if unemployed or fired, less absenteeism at work, reduction of stress and of some physical symptoms or negative emotions, improvement of interpersonal communication. Also Tausczik and Pennebaker (2010) have developed a set of computerized measures or dictionaries to measure some content and stylistic features of language associated with several psychological and interpersonal dimensions, demographic variables, the dominance in the conversation, some basic personality dimensions, proneness to depression and suicide, social bonding after trauma, and lying or truth-telling.

In the present study we sought to investigate the emotion-elaboration processes by applying the linguistic measures developed by Bucci and Pennebaker to writings remotely collected during the confinement period in the Italian Epicenter of COVID-19 Outbreak. Based on the results published in the literature (Lange et al., 2000; Hirai et al., 2020) the expressive writing method proposed online could be a way to promote the health of citizens. However, to the best of our knowledge, the expressive writing technique has never been applied as a method of investigation or intervention during a pandemic outbreak and the consequent confinement at home. We had considered Kacewicz et al.’s (2007) proviso indicating that a technique such as expressive writing may be inappropriate until several weeks or months later a stressful event because people who face a traumatic experience often psychologically distance themselves from the emotional turmoil of the event and this process could be quite healthy in the hours and days after an upheaval. We are also aware that other studies (Range et al., 2000; Stroebe et al., 2002; Bower et al., 2003; Zachariae and O’Toole, 2015) evidenced no effects of writing disclosure when the traumatic experience was uncontrollable, such as a fatal illness or a recent loss. Although these results suggested caution, we considered it useful to test whether the expressive writing technique could be a valid method of investigation and intervention even during the COVID-19 outbreak. In fact, the pandemic and the ensuing confinement, although traumatic, were perhaps not as serious and powerful as the traumas usually investigated in the literature. The state of uncontrollability created by the pandemic may have been less intense than that caused by a fatal illness or a loss. The latter were potential outcomes of the pandemic but not inevitable ones. A previous study (Cohn et al., 2004) conducted in the period before and after the 9/11 terrorist attack had allowed the indicators of emotional change to be effectively detected during that collective tragedy. We therefore set out to conduct a similar study to see if such detection was possible even in the context of the COVID-19 pandemic in the most affected part of the Italian population. Moreover, proposing the expressive writing in an online modality was one of the few ways that adhered to the imposed measures of social distancing while at the same time allowing us to go beyond the global impressionistic evaluations of well-being/distress, and grasp more deeply the subjective processes experienced by people during this critical period.

The aims of the study were: (a) to investigate the main physical, psychological and emotional sensations, and the prevailing themes experienced by the Lombardy population during the confinement period; (b) to detect the eventual changes in emotions, sensations, and themes across the writing sessions; (c) and to test the efficacy of expressive writing in pandemic times by monitoring the emotion elaboration indices from the beginning to the end of the investigation.

## Materials and Methods

### Participants

Sixty-four participants (56 women, 8 men) took part in the online expressive writing project, with ages ranging from 18 to 72 years (*M* = 38.1, *SD* = 15.4).

All but four participants (93.7%) were living in Lombardy during the COVID-19 lockdown period. Twenty-one were single (32.8%), 42 were married or cohabiting (65.6%), and one was divorced (1.6%). During the confinement, 23 were living with their parents (35.9%), 23 were living with a partner and children (35.9%), 17 were living with a partner and no children (26.5%), and one was living alone (1.6%).

The highest level of education obtained was on average quite high: Eight participants (9.4%) had middle school as the highest level of education obtained; 20 (31.3%) had a high school education; 18 (28.1%) a bachelor’s degree; 17 (26.6%) a Master’s degree; and three (4.7%) had a doctorate or similar degree.

Twenty-three participants were students (35.9%); 10 were social and health care professionals (15.6%); 10 were clerical or manual laborers (15.6%); 9 were teachers (14.1%); 6 were retired (9.4%); 3 worked at home (4.7%); and 3 were unemployed (4.7%). Of the 64 participants, one was employed in health care setting dealing with COVID-19 patients, and one had lost her job due to the emergency confinement.

Out of 64 participants, one (1.6%) was a confirmed COVID-19 patient; 37 (57.8%) had no family members or close friends infected with COVID-19; eight (12.5%) had a family member or close friend who had died due to COVID-19; 12 (18.8%) had a family member or close friend hospitalized for COVID-19 symptoms; and six (9.4%) had a family member or close friend infected with COVID-19 but not hospitalized.

### Instruments

#### Expressive Writing Method

Expressive writing is a method created by Pennebaker and Beall (1986) based on the hypothesis that writing about emotional problems can help to improve psychological and physical health of the writer, generating a sense of well-being that can be useful to manage the negative/traumatic experiences and related emotions. Following Pennebaker’s instructions, we gave respondents the following prompt:

Find a time and place where you won’t be disturbed. Ideally, pick a time at the end of your workday or before you go to bed. Promise yourself that you will write for a minimum of 15 min a day for at least 3 or 4 times. You can write every day or less frequently but write at least 1 day a week.

Once you begin writing, write continuously. Don’t worry about spelling or grammar. If you run out of things to write about, just repeat what you have already written. You can write about the same thing on several days of writing or you can write about something different every day. It is up to you.

What to write about: Something that you are thinking or worrying about too much; something that you are dreaming about; something that you feel is affecting your life in an unhealthy way; something that you have been avoiding for days, weeks, or years.

Over the next days, try to write about the deepest emotions and thoughts that you have lived in this period of life. They can be positive or negative, they can concern the present, the past or the future. Whatever you choose to write about, however, it is critical that you let yourself go and explore your very deepest emotions and thoughts.

#### Questionnaire on Personal Data, Emotions, and Physical Symptoms

The questionnaire contained 12 demographic questions: gender, age, job, employment difficulties due COVID-19, voluntary or professional involvement in COVID-19 care settings, place of residence, education, marital status, number and relationship to other people living together with the respondent, and friends or relatives infected by COVID-19. Respondents used a code to track and match their writing submissions.

Afterward a questionnaire proposed by Pennebaker et al. (1990) and Richards et al. (2000) was administered to assess each participant after each writing session. Respondents were asked to rate on a 5-point scale (from 0 – not at all – to 5 – a great deal) the degree to which they had in the past 3 days experienced physical symptoms (e.g., racing heart, upset stomach, headache, dizziness, shortness of breath, cold hands, sweaty hands, and pounding heart), and specified emotions (sad, happy, guilty, altruistic, fearful, brave, proud, humiliated, loved, abandoned, disoriented, suffocated, sacrificed, transgressive, powerful, resigned, angry, and peaceful).

The questionnaire ended with four questions about how the participant felt about their essay that day: “Overall, how much have you told other people about what you wrote today?” “Overall, how much did you reveal your emotions in what you wrote today?” “Overall, how do you feel from a psychological point of view today?” “Overall, how do you feel from a physical point of view today?” The scale for these four questions ranged from 1 to 5.

#### Linguistic Inquiry and Word Count (LIWC)

The Linguistic Inquiry and Word Count (LIWC; Pennebaker et al., 2015) is a software that compares a document to a dictionary of more than 2,300 words and word stems. Each word of the dictionary is assigned to specific linguistic categories; the outputs of the software are percentages of total words of the document associated to each category. The categories of words we examined in this study are as follows:

- Emotion-related words (Positive sensations, Positive emotions, Optimism, Negative emotions, Sadness, Anger, and Anxiety).
- Cognitive process words (Causation, Introspection, Inhibition, Self-discrepancies, Possibility, and Certainty).
- Time markers (Past tenses, Present tenses, and Future tenses).
- Current concerns (Movement, Occupation, Work, School, Achievement, Leisure, Home, Sport, TV, Music, Money, Metaphysical issues, Religion, Death, Physical functions, Body, and symptoms, sexual issues, grooming, eating, and sleep).

#### Discourse Attributes Analysis Program (DAAP)

The Italian Discourse Attribute Analysis Program (Italian DAAP; Bucci and Maskit, 2006; Maskit and Murphy, 2011; Mariani et al., 2013; Negri et al., 2018) compares any kind of Italian text with word lists or dictionaries; the output is a list of counts and indices indicating the proportion in which those words are present in the texts examined or the average weight of words in respect to a certain construct, weight previously empirically assigned to each word present in the dictionaries. For this study we applied the following Italian dictionaries or linguistic measures of the referential process:

- *IAffN*: Italian dictionary of negative affects; it provides the proportion of words in the text related to negative affects.
- *IAffP*: Italian dictionary of positive affects; it provides the proportion of words in the text related to positive affects.
- *IREF*: Italian dictionary of refection-related words; it provides the proportion of words in the text referring to cognitive or logical functions, and to communication processes that imply the use of cognitive functions;
- *IWRAD:* The Italian Weighted Referential Activity Dictionary allows to define for each analyzed text the average of the weights that the words assume in terms of referential activity (i.e. weights related the degree of concreteness, specificity, clarity and imagery). The referential activity in fact can be defined as the degree to which the speaker or writer is able to translate their emotional, visceral and relational experience into words, so as to evoke corresponding experiences in the listener or in the reader (Bucci et al., 1992). It is a measure of emotional involvement and the connection between words and the emotional experience. IWRAD scores range from 0 (lowest) to 1 (highest RA).
- *HPIWRAD*: High Proportion Italian WRAD is the proportion of texts examined that has a WRAD above the average value.
- *IWRRL*: The Italian Weighted Reflection and Reorganization List is a dictionary of Italian words associated to the Reflection and Reorganization function. This can be defined as the degree to which the speaker is trying to recognize and understand the emotional significance of an event or set of events in their own or someone else’s life, or in a dream or fantasy. IWRRL is an index of personal elaboration of emotional experiences; IWRRL scores range from 0 (lowest) to 1 (highest RR).
- *IWRAD_IWRRL*: It is the covariation in the texts of WRAD and WRRL measures. A positive covariation generally indicates a good elaboration process since the emotional involvement in the storytelling indicated by WRAD is associated with a personal and not abstract reflection on this emotional activation.

### Procedure

The aims of the study were presented thorough a 3-page website entitled “#IStayAtHomeAndWrite.” In the first page there was an invitation to participate in a study designed to explore how people were managing the psychological emergency produced by the COVID-19 outbreak. It was specified that whoever participated would not receive particular advice or recommendations, but would instead, potentially activate and enhance their own personal resources already at their disposal for facing the emergency and confinement period. In order to engage and motivate potential respondents, the second page reported a detailed and documented list of benefits derived from the expressive writing method. The third page presented the expressive writing instructions as proposed by Pennebaker et al. (1988): that is, when and how much to write, what to write about, and what to do with the writing samples. The page contained a link to an online form with the questionnaire, and a field in which to enter the personal writing required. Respondents could choose to fill out anonymously the form or, if they wanted, to receive an automated report of what they had written by entering an email address. The link to the website was circulated mainly through social networks, websites of the local community (as local library websites), general practitioners’ mailing lists and word of mouth. We excluded from participation one person who entered two writings, each composed of just one nonsense word.

### Writings Collected

Due the COVID-19 outbreak the Italian Government had decreed a mandatory confinement at home for all resident population for 8 weeks, form March 9 to May 4, 2020. It was possible to leave the home only for serious health reasons or for work, and only if performing in a few essential jobs. During this period, we launched the expressive writing project, collecting a total of 167 writings from 64 respondents. The average length of writing was 1031 words (*SD* = 1187; min = 38, max = 6267). Thirty-one respondents (48.4%) sent only one writing sample, nine respondents (14.1%) sent two writings; ten respondents (15.6%) sent 3 writings; four respondents (6.3%) four writings; four respondents (6.3%) five writings; one respondent (1.6%) six writings; two respondents (3.1%) seven writings; two respondents (6%) eight writings; and one respondent (1.6%) sent 14 writings. Regarding the distribution of the writings along the 8 weeks of confinement, 49 texts (29.3%) were written in the third week; 45 (26.9%) were written in the fourth week; 31 (18.6%) in the fifth week; 19 (11.4%) in the sixth week; 13 (7.8%) in the seventh week; and 10 (6.0%) in the eighth week.

### Statistical Analyses

To test for significant differences between groups of respondents we ran a series of analysis of variance (ANOVA); to detect significant differences between measurements of the same variables at different times we ran a series of repeated measures ANOVAs, as well as to test different but similar measurements on the same participants (e.g., for ratings about the various emotions perceived or about psychological and physical well-being); to test a causal relationship between different variables, we ran a series of linear regression analyses; finally, to summarize the ratings on physical sensations, those on perceived emotions, and those on the themes emerging in the texts, we conducted a series of exploratory factorial analyzes, using the “minimum residual” extraction method in combination with a “varimax” rotation.

## Results

### Physical Sensations

After each writing session, respondents were asked to rate on a scale ranging from 1 (very bad) to 5 (very well) how they felt from a psychological and physical point of view. On average, physical well-being was significantly higher than psychological [*F*_(1, 63)_ = 13.02, *p* < 0.001, η^2^*_*p*_* = 0.173] and the average score exceeded the neutral value indicating a prevalence of a feeling of physical well-being (*M* = 3.25, *SD* = 0.88). Physical well-being scores did not vary by age, education, profession, total number of writing sessions held, and also with respect to being among close friends or relatives who either had been infected or deceased from COVID-19. There was also no difference in physical well-being between the first and last writing session.

**FIGURE 1:**
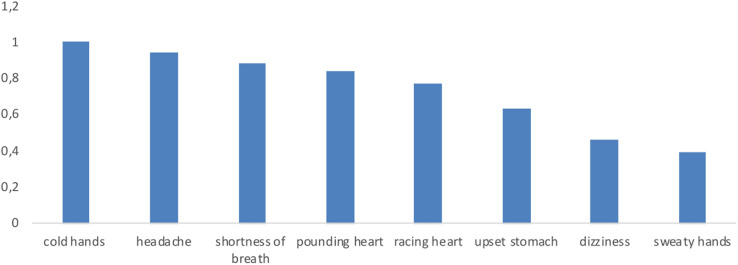
Respondents’ physical sensations and symptoms average ratings.

Overall, the participants reported very little unpleasant physical sensations (see Figure 1; the means ranged from 0.70 to 1.09 on a 5-points scale); among those they experienced, headaches and cold hands prevailed and were significantly higher than the sensation of sweaty hands [*F*_(7, 441)_ = 6.31, *p* < 0.001, η^2^*_*p*_* = 0.091]. The cold hands sensation was even greater in respondents with at least one confirmed or deceased COVID-19 patient among their close friends or family members [*F*_(__1__)_ = 6.14, *p* = 0.028, η^2^ = 0.075] along with shortness of breath [*F*_(__1__)_ = 4.51, *p* = 0.038, η^2^ = 0.068]. The sensation of cold hands finally decreased significantly between the first and the last writing session [*F*_(1, 32)_ = 5.58, *p* = 0.024, η^2^*_*p*_* = 0.148].

### Emotions

#### Self-Reported Emotions

As previously mentioned, perceived psychological well-being was overall lower than the physical well-being [*F*_(1, 63)_ = 13.02, *p* < 0.001, η^2^*_*p*_* = 0.173], with average score close to neutral value “neither well nor bad” (3) values (*M* = 2.94, *SD* = 0.72). None of the variables (age, educational qualification, profession, total number of writing sessions held, and the presence among close friends and relatives of at least one confirmed or deceased COVID-19 patient) had any effect on self-reported emotions. There was also no difference in psychological well-being between the first and last writing session.

Analyzing the emotions felt by respondents during the days of confinement we observed a well-defined and consistent profile both in self-reported evaluation and in the emotions emerging from writings.

**FIGURE 2:**
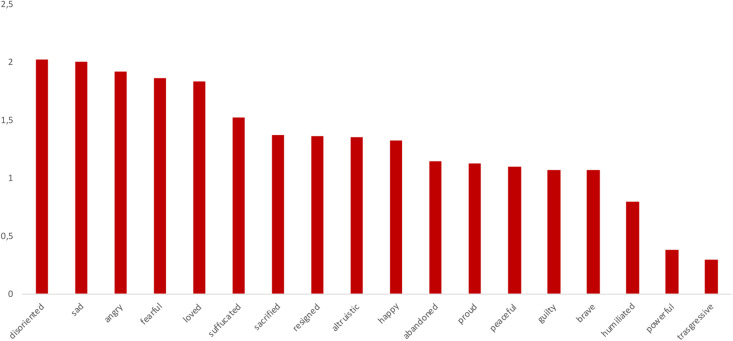
Respondents’ emotions average ratings.

The emotions that respondents rated higher were “sad” (*M* = 2.05, *SD* = 0.90), “disoriented” (*M* = 2.04, *SD* = 1.07), “angry” (*M* = 1.94, *SD* = 1.13), “fearful” (*M* = 1.92, *SD* = 1.09), “loved” (*M* = 1.87, *SD* = 1.07), and “suffocated” (*M* = 1.59, *SD* = 1.20). Emotions like “powerful” (*M* = 0.40, *SD* = 0.65) and “transgressive” (*M* = 0.38, *SD* = 0.68) were almost absent (see Figure 2); a significant difference was found between the most present and the least present emotions [*F*_(17, 1071)_ = 19.8, *p* < 0.001, η^2^*_*p*_* = 0.239].

The younger respondents (18–25 y) felt significantly sadder than older ones (51–72 years) [*F*_(1)_ = 4.89, *p* = 0.004, η^2^ = 0.196]; those who had a confirmed infection or a deceased COVID-19 patient among their relatives or close friends felt much more fearful than those who did not [*F*_(1)_ = 4.23, *p* = 0.044, η^2^ = 0.064]. In the last writing session, the respondents felt less fearful [*F*_(1, 32)_ = 5.71, *p* = 0.023, η^2^*_*p*_* = 0.152] but also less altruistic [*F*_(1, 32)_ = 5.45, *p* = 0.026, η^2^*_*p*_* = 0.145] and happy [*F*_(1, 32)_ = 5.40, *p* = 0.027, η^2^*_*p*_* = 0.144].

Exploratory factor analysis on self-rated emotions revealed two dimensions, or two sets of emotions that tend to be felt together (see Table 1): a negative emotion factor (28.4% explained variance) and a prosocial emotions factor (18.9% explained variance). Both factors decreased from first to last writing session [negative emotions: *F*_(1, 32)_ = 5.08, *p* = 0.031, η^2^*_*p*_* = 0.137; prosocial emotions: *F*_(1, 32)_ = 4.89, *p* = 0.034, η^2^*_*p*_* = 0.133].

**TABLE 1:** Exploratory Factor Analysis on self-reported emotions.

	**Negative emotions**	**Prosocial emotions**	**Uniqueness**
Suffocated	**0.765**	–0.151	0.391
Sad	**0.763**	–0.158	0.392
Fearful	**0.737**	0.226	0.404
Disoriented	**0.732**	0.200	0.423
Abandoned	**0.678**	–0.024	0.539
Angry	**0.664**	–0.023	0.558
Scarified	**0.619**	–0.108	0.605
Humiliated	**0.576**	–0.116	0.654
Resigned	**0.557**	–0.130	0.672
Guilty	**0.529**	0.110	0.708
Altruistic	0.161	**0.802**	0.330
Brave	0.097	**0.768**	0.400
Proud	–0.021	**0.768**	0.409
Happy	–0.172	**0.629**	0.575
Loved	–0.054	**0.592**	0.646
Powerful	–0.090	**0.498**	0.743

*“Minimum residual” extraction method was used in combination with a “varimax” rotation. In bold the factor loadings of the items retained in each factor.*

#### Emotions Emerging in Writings

To the question “how much did you reveal your emotions in what you wrote today?” the respondents’ rating was between “somewhat” (3) and “much” (4) (*M* = 3.16, *SD* = 1.15). The writings thus were considered emotion-laden by those who wrote them. This was even more marked for those who wrote at least three times consecutively (*M* = 3.56, *SD* = 0.99), as instructions required, compared to those who wrote only once or twice [*M* = 2.92, *SD* = 1.18; *F*_(1)_ = 4.80, *p* = 0.032, η^2^ = 0.072].

The presence of emotions in the writings is confirmed by the computerized analysis through both the DAAP and LIWC dictionaries. Words referring to emotions were 5.02% for DAAP and 5.46% for LIWC; Negative affects (2.43%) prevailed over positive affects (1.95%) and over neutral affects (0.6%) for DAAP [*F*_(2, 126)_ = 5.08, *p* = 0.031, η^2^*_*p*_* = 0.137]. Negative emotions (2.98%) prevailed over positive emotions (0.82%) for LIWC as well [*F*_(1, 63)_ = 91.7, *p* < 0.001, η^2^*_*p*_* = 0.593]. In particular, sadness (1.26%) prevailed over anxiety (0.81%) and anger (0.56%) [*F*_(2, 126)_ = 16.6, *p* < 0.001, η^2^*_*p*_* = 0.209].

Respondents with at least a close friend or a relative with confirmed infection or deceased from COVID-19 produced writings with less positive emotions (DAAP) compared to those without infected relatives and friends [*F*_(1)_ = 4.56, *p* = 0.037, η^2^ = 0.068], however, the two groups did not differ in negative emotion words. No significant variations were found in emotion-related words between first and last writing session.

### Themes

**FIGURE 3:**
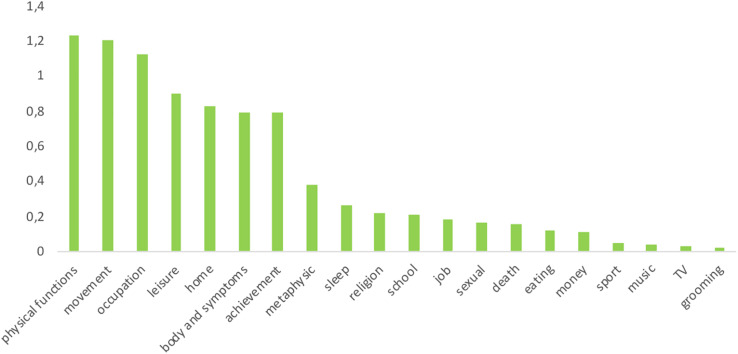
Themes emerging from respondents’ writings.

Considering all writings, present time markers were more prevalent than past or future time markers [*F*_(2, 126)_ = 16.6, *p* < 0.001, η^2^*_*p*_* = 0.209]. In regards to themes (see Figure 3), words related to movement, leisure, home, body and symptoms, physical functions, achievement, and occupation were much more present than words related to school, job, sport, TV, music, money, metaphysic, religion, death, sexual, sleep, eating, grooming [*F*_(19, 1197)_ = 57.3, *p* < 0.001, η^2^*_*p*_* = 0.476].

An exploratory factor analysis revealed four dimensions (see Table 2) or themes: a pleasant theme factor (17.9% explained variance), an existential theme factor (17.2% explained variance), a bodily theme factor (15.6% explained variance), and a duty theme factor (17.2% explained variance). Both pleasant and duty themes factors decreased from first to last writing session [pleasant themes: *F*_(1, 32)_ = 3.39, *p* = 0.050, η^2^*_*p*_* = 0.096; duty themes: *F*_(1, 32)_ = 5.02, *p* = 0.032, η^2^*_*p*_* = 0.136].

**TABLE 2:** Exploratory factor analysis on themes emerging from writings.

	**Factor**				**Uniqueness**
	**Pleasant**	**Existential**	**Bodily**	**Duty**	
Leisure	**0.982**	–0.036	–0.086	0.082	0.019
Home	**0.966**	–0.018	–0.097	0.083	0.049
Metaphysic	–0.031	**1.039**	–0.034	–0.075	–0.089
Religion	–0.043	**0.796**	–0.103	–0.102	0.342
Death	0.006	**0.395**	0.098	–0.015	0.833
Physical	–0.007	0.015	**1.029**	–0.022	–0.060
Body	–0.030	0.036	**0.672**	–0.003	0.545
Sleep	–0.075	–0.005	**0.402**	–0.004	0.832
Occupation	0.241	–0.083	–0.055	**0.982**	–0.033
Job	0.008	0.036	–0.005	**0.574**	0.668
School	–0.009	–0.090	0.009	**0.353**	0.867

*“Minimum residual” extraction method was used in combination with a “varimax” rotation. In bold the factor loadings of the items retained in each factor.*

### Emotional Elaboration

Expressive writing appeared to significantly facilitate the processing of emotions as evidenced by multiple linguistic markers measured by the DAAP as well as the LIWC measures.

First, respondents considered what they had written as something quite personal and when asked “overall, how much have you told other people about what you wrote today?” they gave an average score close to 2 (little) value (*M* = 2.33, *SD* = 1.17). As mentioned above, participants tended to consider their writings as emotionally charged (*M* = 3.16, *SD* = 1.15).

If we consider all the writings together, the referential activity and the reflection/reorganization activity was above average value (IWRAD = 0.505, *SD* = 0.006; IWRRL = 0.551, *SD* = 0.004). The proportion of text in respondents’ writings with referential activity above the average value was very high (HPIWRAD = 0.68 corresponding to 68% of textual corpus, *SD* = 0.20) and the covariation between referential activity and reflection/reorganization activity was on average positive (IWRRL_IWRAD = 0.39, *SD* = 0.37). The percentage of words related to cognitive processes (causation, introspection, discrepancy, inhibition, possibility, certainty) as detected by the LIWC was equal to 5.47% (*SD* = 1.69), and words related to the abstract reflection, as measured by DAAP, was equal to 0.03 (*SD* = 0.01) corresponding to 3% of total number of words.

The writings of those who wrote following the instructions – write at least three times – compared to those who wrote at most once or twice, were much longer [*F*_(1)_ = 37.1, *p* < 0.001, η^2^ = 0.374].

Emotional processing indexes were significantly better for those who had a confirmed infected or dead COVID-19 patient among their relatives or close friends, than those who did not. They had lower abstract reflection [*F*_(1, 62)_ = 7.81, *p* = 0.007, η^2^*_*p*_* = 0.112]; higher –although marginally significant– referential activity [*F*_(1, 62)_ = 3.56, *p* = 0.064, η^2^*_*p*_* = 0.054], and the covariation between referential and reflection/reorganization activities [*F*_(1, 32)_ = 3.74, *p* = 0.058, η^2^*_*p*_* = 0.057]. These differences mean that having a relative or close friend infected or deceased from COVID-19 predicted almost significantly the emotional processing indexes in the respondents’ writings [IRef: *R*^2^ = 0.112, *F*_(1, 62)_ = 7.81, *p* = 0.007, *t* = −2.80, *p* = 0.007; IWRAD: *R*^2^ = 0.054, *F*_(1, 62)_ = 3.56, *p* = 0.064, *t* = 1.89, *p* = 0.064; IWRAD_IWRLL: *R*^2^ = 0.057, *F*_(1, 62)_ = 3.74, *p* = 0.058, *t* = 1.93, *p* = 0.058].

The results are even more significant when taking into account the level of psychological well-being perceived by the respondents. Lower levels of perceived psychological well-being predicted higher levels of emotional processing scores in writings [IWRRL: *R*^2^ = 0.135, *F*_(1, 54)_ = 8.43, *p* = 0.005, *t* = −2.90, *p* = 0.005; Cognitive Processes: *R*^2^ = 0.079, *F*_(1, 54)_ = 4.67, *p* = 0.035, *t* = −2.16, *p* = 0.035; Introspection: *R*^2^ = 0.112, *F*_(1, 54)_ = 6.84, *p* = 0.012, *t* = −2.61, *p* = 0.012; Inhibition: *R*^2^ = 0.095, *F*_(1, 54)_ = 5.66, *p* = 0.021, *t* = −2.38, *p* = 0.021].

In regard to themes, existential themes (existential themes factor) correlated positively with IWRAD (*r* = 0.568, *p* < 0.001) and WRAD_WRRL covariation (*r* = 0.257, *p* < 0.05). When respondents wrote about death, religion and, more generally, about existential themes, the indices of emotional engagement and emotional processing tended to increase.

**FIGURE 4:**
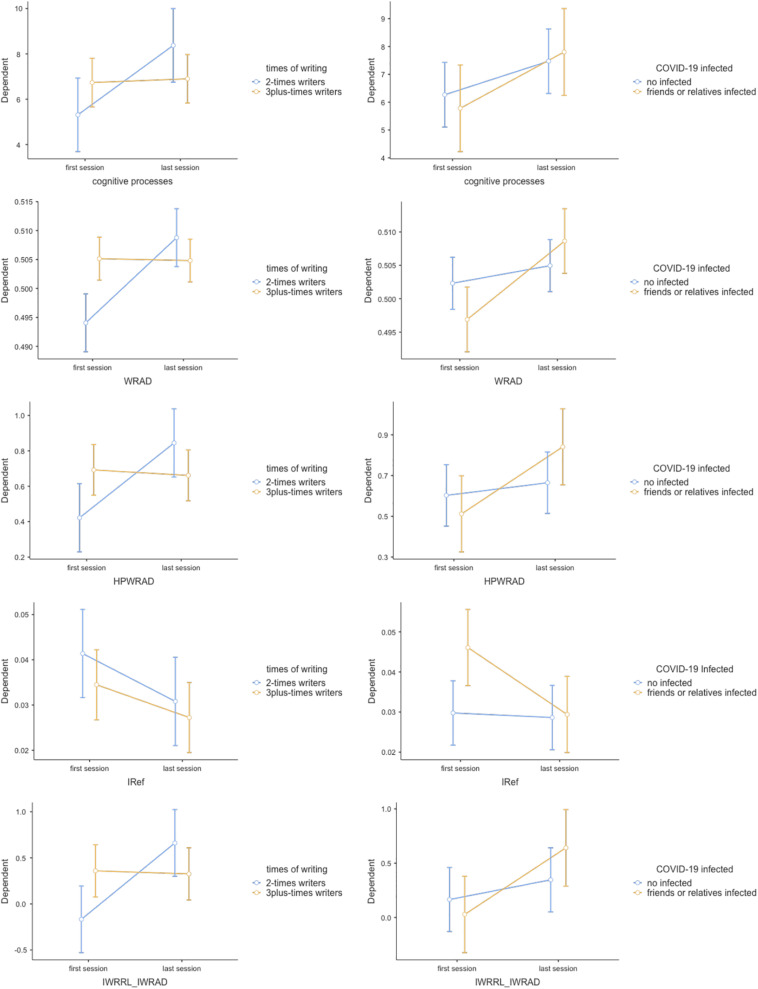
Estimated marginal means from repeated measures ANOVAs between first and last writing sessions – interaction effects about “Emotion-elaboration-indices” × “COVID-19 infected” × “Times-of-writing” model.

The effect of repeating the writing was multifaceted. When we compared the first session writings with those of the last session, we did not find any significant difference in the indices of emotional processing. When instead we computed in the analyses the comparison between those who wrote only 2 times (2-times writers) with those who wrote at least 3 times (3plus-times writers), and between those who had a family member or close friend infected or deceased form COVID-19 with those who had not, then significant interaction effects on the changes between the first and the last writings were found. In particular between first and last session of writing we found the following significant interaction effects (see Figure 4):

a) cognitive processes (as measured by LIWC, both globally as well as individually considered) increased only in 2-times writers [cognitive processes: *F*_(1, 29)_ = 6.18, *p* = 0.019, η^2^*_*p*_* = 0.166; cognitive processes x 2/3plus-times writers: *F*_(1, 29)_ = 4.50, *p* = 0.042, η^2^*_*p*_* = 0.127];
b) the referential activity increased much more in 2-times writers, especially in those with an infected or dead CODIV-19 patient among close friends or relatives than in the opposite group [IWRAD: *F*_(1, 29)_ = 10.64, *p* = 0.003, η^2^*_*p*_* = 0.269; IWRAD x Infected; *F*_(1, 29)_ = 4.27, *p* = 0.048, η^2^*_*p*_* = 0.128; IWRAD x 2/3plus-times writers: *F*_(1, 29)_ = 11.63, *p* = 0.002, η^2^*_*p*_* = 0.286];
c) the text proportion with high referential activity increased only in 2-times writers [HPIWRAD: *F*_(1, 29)_ = 5.40, *p* = 0.027, η^2^*_*p*_* = 0.157; HPIWRAD x 2/3plus-times writers: *F*_(1, 29)_ = 7.26, *p* = 0.012, η^2^*_p_* = 0.200];
d) the abstract reflection decreased only in responders with friends or relatives COVID-19 infected [IRef: *F*_(1, 29)_ = 5.20, *p* = 0.030, η^2^*_p_* = 0.152; IRef x Infected: *F*_(1, 29)_ = 3.97, *p* = 0.050, η^2^*_*p*_* = 0.120];
e) the covariation between IWRAD and IWRRL increased only in 2-times writers [IWRAD_IWRRL: *F*_(1, 29)_ = 7.15, *p* = 0.012, η^2^*_*p*_* = 0.198; IWRAD_IWRRL × 2/3plus-times writers: *F*_(1, 29)_ = 8.38, *p* = 0.007, η^2^*_*p*_* = 0.224].

## Discussion

The corpus of texts we collected thorough an online expressive writing project presents an overall picture of subtle but pervasive change in linguistic markers of emotional elaboration during COVID-19 outbreak in Lombardy, the Italian region that first was affected by the pandemic in Europe, and has had the highest number of deaths and confirmed cases in Italy.

First of all, from our results we see that that despite the upheavals of the COVID-19 outbreak and of the sudden and unprecedent experience of confinement, which lasted 8 weeks, participants maintained a very good level of perceived physical well-being and quite good perceived psychological well-being. The health and psychological emergencies appear to have mobilized a person’s resources and resilience to face the new threat. A similar effect was registered during the 9/11 terrorist attack where it was observed that the majority of the population maintained a relatively high level of wellbeing despite the upsetting situation (Bonanno et al., 2006). Probably the fact that all respondents were living during confinement with their relatives provided enough support to maintain a sense of control and to foster their resilience (Yang and Ma, 2020).

The profile of emotions experienced by respondents highlighted a positive side of their emotional reaction. The negative emotions prevailed, first of all sadness – especially in younger participants – followed immediately by fear – especially in respondents with friends or relatives who had been COVID-19 infected – and then followed by disorientation, and anger. Alongside these emotions, however, respondents experienced – albeit with less intensity – a parallel set of emotions, such as being loved, feeling altruistic, proud, happy, and brave. Both negative and positive emotions significantly decreased as the writing sessions followed.

A similar manifold profile results from the analysis of themes. Writings developed around four thematic areas: pleasant (home and leisure), existential (metaphysic, religion, death), bodily (physical, body, and sleep), and duty themes (occupation, job, and school). Duty and pleasant ones decreased over writing sessions.

Perhaps the most important findings are in regard to the linguistic measures of emotion processing. We sought to understand whether the online expressive writing task helped respondents to get more in touch with the intense emotions experimented following the upheavals they witnessed. Multiple results from our study support an affirmative answer to this question.

First of all, respondents on average reported that they had revealed their emotions to a considerable extent in their writings, while they considered to have only partially talked to other people about what they wrote. These evaluations were even more marked in the respondents who followed the instructions completely, i.e., writing at least three times on consecutive days. A sign of their greater involvement was also evidenced by the length of their writings, which was lengthier than those who wrote only once or twice.

The linguistic analysis of all the writings confirmed the full emotional involvement of the respondents and their ability to narrate in a vivid, clear, specific and concrete way their emotional experience. In terms of Bucci’s Multiple Code Theory, their stories had a level of referential activity above average (see IWRAD and HPIWRAD). They were also able to develop personal reflections on the emotional meaning of what they were writing and experiencing, thus activating a process of reorganization of the experience and a capacity for introspection (see WRRL, IWRAD_IWRRL, LIWC cognitive processes).

Analyzing the between-subjects differences, the respondents who had a friend or a relative who was confirmed to be infected or had passed away from COVID-19, showed the highest emotional engagement and emotional processing values. Compared to the others, they had higher referential activity, lower abstract reflection and more positive covariation between referential and reflection/reorganization activities, indicating a better emotional elaboration. Their experience was certainly more threatening and potentially traumatic. In fact, they were prevented from visiting their loved ones if they were hospitalized, from accompanying them in the last moments of their life and from taking part in their funerals. In these cases, the writing protocol appears to have allowed an increased expression and elaboration of intense emotions.

Another finding along these lines is the high positive correlation between themes related to death, religion and more generally to existential aspects on the one hand, and referential activity and its covariation with reflection/reorganization activity on the other. It is precisely when they wrote about these topics that respondents showed an increase in these indices of emotional engagement and elaboration.

Also, the level of perceived psychological well-being had an impact on the emotional processing indexes. The more the person perceived low psychological well-being, the more the reflection/reorganization activity and, more generally, the cognitive processes activated in writing increased.

Lastly, we found that the improvement of the emotional processing indices between the first and the last writing session applied only to those participants who had had at least a COVID-19 infected or deceased patient among close friends or relatives and to those participants who wrote only twice. Analyzing the average values of those who wrote at least three times and those who did not have a friend or relative infected from COVID-19, it is noted that the emotional processing values were already high in the first writing session and remained so over all sessions. These data are important in that they help us understand to what extent and for whom the expressive writing method could be useful in times of pandemic. It is likely that participants who wrote at least three times were already used to writing about themselves or more generally to reflecting on their emotions. For them, the expressive writing experience allowed them to be more in touch with their emotions. By contrast, those who wrote only twice had a significant emotional activation from first to second writing session. For these participants this high emotional activation probably led to the interruption of the task, which may have been too intense to be expressed and processed during the writing period. Some studies (Jensen-Johansen et al., 2018; Renzi et al., 2020), in fact, evidenced that the degree of people’s emotional regulation ability have a moderating effect on writing disclosure effectiveness. People who score too low or too high in emotion regulation ability found it difficult to benefit from expressive writing. We can therefore hypothesize that those who interrupted the writing task did so due to difficulties in regulating the emotions activated by it. However, those of this group who wrote even only twice saw an improvement in the indices of emotional elaboration. Lastly, the expressive writing method proved to be particularly useful for participants who had at least a COVID-19 infected or deceased patient among close friends or relatives. In fact, in their writings there was an increase in both the emotional involvement and the ability to reflect and give meaning to the challenging emotional experience.

In sum, on the basis of these findings we can argue that the online expressive writing experience can be useful in situations of psychological emergency such as that of a pandemic, and that writing even just once or twice is particularly useful for those who are more directly in contact with situations of contagion and death. However, for some people – especially those with difficulty in emotion regulation – the task of writing about themselves can be too demanding and challenging. For these people, expressive writing could probably be of greater benefit if inserted in a context of support and containment such as that represented by the relationship with a clinician.

Our findings are consistent with those of Cohn et al. (2004). They investigated the linguistic markers of the psychological change surrounding the 9/11 terroristic attack analyzing the diaries of 1,084 U.S. users of an on-line journaling service. They also found an increase in negative emotions and cognitive processes related to the upsetting event and a subsequent decrease in the following days and months. However, they also registered an increase in social orientation (namely how often participants used words such as talk, share, or friends and personal pronouns other than first-person singular) and psychological distancing (namely articles and words of more than six letters and inverse scores for first-person singular pronouns, words indicating discrepancy from reality, and present-tense verbs) that we did not observe. We believe that this difference is due to the fact that in our case the participants did follow a real expressive writing protocol differently from Cohn and colleagues’ study where diaries were posted to be read online. Moreover, the different nature of the threats that the writers were experiencing in the two different contexts may also have played a role. The linguistic analyses of our respondents’ writing have showed that expressive writing in times of an upheaval, such as a pandemic, can lead to a significant emotional engagement and elaboration.

A limitation of our study includes the fact that our participants were not a casual representative sample and thus results may differ from those of the general population. Furthermore, more than half of the participants did not follow the instructions in full, and wrote only once or at most twice; so, those who accepted our invitation to participate in the project, and wrote at least three times, were perhaps already used to writing or thinking about themselves; therefore, it remains to be seen if expressive writing is an effective tool for everyone. Secondly, differently from other studies on the expressive writing efficacy, we cannot plan follow-up measurements; also, in our study we do not have an external criterion of improvement, such as measures of behavior change or physiological indexes of wellbeing. Thirdly, mainly for ethical reasons, we have not involved a control group which could have highlighted if the observations related to the writing process could be linked to time passage, independently from the writing intervention.

Notwithstanding the above limitations, this project offers fresh insights into how people respond psychologically to large-scale upheavals that affect large populations. In particular, our results indicate that expressive writing can be used in the context of a psychological emergency, both as a powerful instrument to investigate and detect the complex psychodynamic processes underpinning the distress, and as a useful intervention to reduce the negative impact of traumatic events.

## Data Availability Statement

The data set supporting the conclusions of this article will be made available by the authors, without undue reservation, to any qualified researcher.

## Ethics Statement

The present study involving human participants was reviewed and approved by the Ethics Committee, University of Bergamo. The patients/participants provided their written informed consent to participate in this study.

## Author Contributions

AN contributed to the research design ideation, online instruments (website and form) setting, statistical analyses, and manuscript writing. GA contributed to the online instruments setting, statistical analyses, and manuscript writing. AB contributed to the statistical analyses and manuscript writing. CZ contributed to the online instruments setting and manuscript writing. CC contributed to the research design ideation, online instruments setting, manuscript writing, and English editing. All authors contributed to the article and approved the submitted version.

## Conflict of Interest

The authors declare that the research was conducted in the absence of any commercial or financial relationships that could be construed as a potential conflict of interest.
